# How Resilient is Wood Xylan to Enzymatic Degradation in a Matrix with Kraft Lignin?

**DOI:** 10.1021/acs.biomac.4c00185

**Published:** 2024-05-15

**Authors:** Jana B. Schaubeder, Christian Ganser, Tiina Nypelö, Takayuki Uchihashi, Stefan Spirk

**Affiliations:** † Institute of Bioproducts and Paper Technology (BPTI), Graz University of Technology, Inffeldgasse 23, 8010 Graz, Austria; ‡ Exploratory Research Center on Life and Living Systems, National Institutes of Natural Sciences, 5-1 Higashiyama, Myodaiji, 444-8787 Okazaki, Japan; § Department of Bioproducts and Biosystems, Aalto University, Vuorimiehentie 1, 02150 Espoo, Finland; ∥ Chalmers University of Technology, Kemivägen 10, 41296 Gothenburg, Sweden; ⊥ Department of Physics, Nagoya University, Chikusa-ku, Furo-cho, 464-8602 Nagoya, Japan

## Abstract

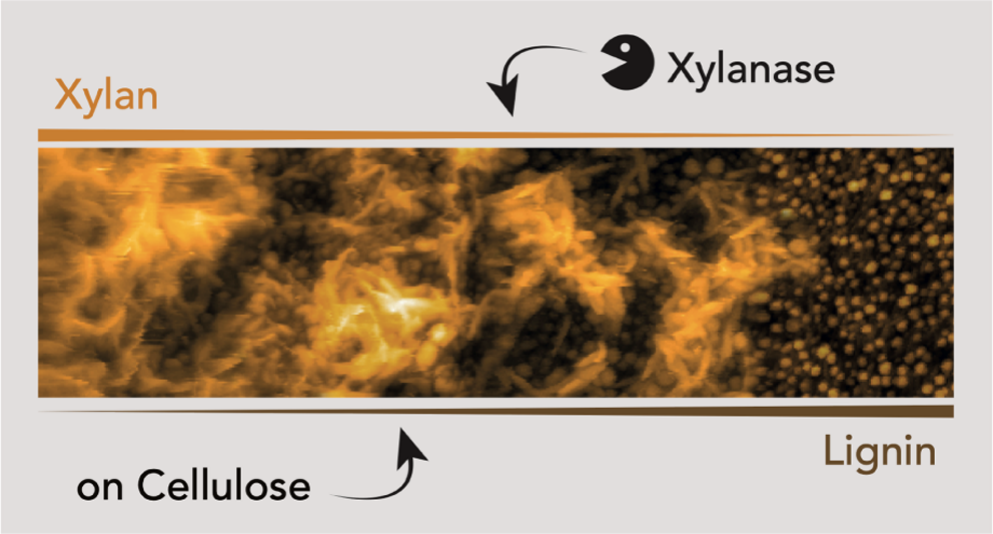

Despite the potential of lignocellulose in manufacturing value-added chemicals and biofuels, its efficient biotechnological conversion by enzymatic hydrolysis still poses major challenges. The complex interplay between xylan, cellulose, and lignin in fibrous materials makes it difficult to assess underlying physico- and biochemical mechanisms. Here, we reduce the complexity of the system by creating matrices of cellulose, xylan, and lignin, which consists of a cellulose base layer and xylan/lignin domains. We follow enzymatic degradation using an endoxylanase by high-speed atomic force microscopy and surface plasmon resonance spectroscopy to obtain morphological and kinetic data. Fastest reaction kinetics were observed at low lignin contents, which were related to the different swelling capacities of xylan. We demonstrate that the complex processes taking place at the interfaces of lignin and xylan in the presence of enzymes can be monitored in real time, providing a future platform for observing phenomena relevant to fiber-based systems.

## Introduction

The efficient conversion of biomass remains a challenge as it is inherently resilient to enzymatic hydrolysis in order to withstand attacks by microorganisms.^1−3^ Furthermore, it is known that some components of the biomass, such as lignin, even have a negative effect on enzymatic degradation (i) by limiting the accessibility of enzymes and (ii) by temporarily inactivating enzymes via nonspecific interactions, thereby reducing the efficiency of the enzymes.^4−6^ Although the interactions between lignin and cellulases and the influence of lignin on the degradation of cellulose have been extensively studied,^7−10^ the role of hemicellulose in the overall process is largely neglected. A limitation has been the challenge of experimentally investigating the phenomenon directly in the biomass substrate due to their complexity and accessibility to real-time methods, requiring the use of simplified models with all their pros and cons.^11−14^ Particularly, lignocellulose thin films have been extensively used by many researchers in the past two decades to explore interactions taking place on the interface. This also included the use of lignocellulose degrading enzymes, which have been applied on either pure (i.e., only one polymeric constituent of the cell wall) or mixtures (i.e., two or more lignocellulose components). These thin films feature a two-dimensional structure, as only the thickness of the films changes upon treatment but not their lateral dimensions.^15^ Owing to their simpler morphology, more sophisticated analytical techniques become available, such as high-speed atomic force microscopy (HS-AFM) and surface plasmon resonance spectroscopy (SPRS). HS-AFM enables real-time imaging with a high resolution in liquids. In addition, it is a force-dependent method that allows for drawing conclusions about the mechanical behavior (hardness and softness) of the investigated materials. SPRS, on the other hand, is a real-time monitoring method enabling the association and dissociation of molecules on surfaces, thereby providing insights into binding kinetics and dynamics. The high-resolution images obtained by HS-AFM provide information about the structural and mechanical properties of biomolecules and can be combined with the binding kinetics and affinities between biomolecules from SPRS measurements. In this way, more thorough understanding of biomolecular interactions can be obtained than by using only one of these methods.^16^

The present work focuses on finding out how resilient is xylan to enzymatic degradation when it is associated in a layer with lignin. Therefore, we created a matrix containing the polymeric parts of the cell wall, allowing for the analysis of enzymatic breakdown processes. In detail, we qualitatively and quantitatively determined the degradation efficiency of an endoxylanase for xylan at varying lignin contents by HS-AFM and SPRS.

## Materials and Methods

### Materials

Xylan isolated from birchwood (DP 70-1000, xylose content ≥90% by high-performance anion exchange (HPAE), product code X0502) was purchased from Sigma-Aldrich and used without further purification.^17^ The used xylan contained approximately one uronic acid unit per 11 xylose units.^18^ Lignin was obtained from Sigma-Aldrich (lignin, kraft, from pine) and used without further purification. The used lignin had a molecular mass of 1.9 kDa (*M*_n_ 1.87 kDa, *M*_w_ 7.07 kDa, *M*_z_ 24.6 kDa, PDI 3.78), determined by gel permeation chromatography with an size-exclusion chromatography (SEC) (WGE Dr. Bures GmbH, 112 μL injection volume, A2 = 0.5 μL mol·g^–2^, duration 30 min, eluent tetrahydrofuran (THF), and flow rate 1.0 mL·min^–1^). For the separation, two 5 μm MZgel-SDplus linear columns (MZ Analyzetechnik) equipped with a refractive index and a UV detector were used. To dissolve the lignin in THF, it was acetylated prior to the measurement by mixing acetic acid anhydride (2.5 equiv for full conversion with respect to the hydroxyl groups in the dry lignin sample; 10 mmol·g^–1^ lignin_dry_) in a 1:1 molar ratio with pyridine. The reaction was supported by microwave-assisted heating using a Monowave 50 apparatus (Anton Paar, Austria), holding a temperature of 150 °C for 1 min. The reaction was quenched with 30 mL of 0.1 M HCl, filtered, and washed with water and HCl. The brown filter cake was dried at 50 °C (74% yield based on the lignin). Sodium phosphate monobasic dehydrate (purum p.a., crystallized, ≥99.0%), sodium phosphate dibasic dehydrate (BioUltra, ≥99.0%), and bovine serum albumin (BSA, lyophilized powder ≥96%, A2153) were purchased from Sigma-Aldrich. Chloroform (CHCl_3_, stabilized with about 0.6% ethanol) and hydrochloric acid (HCl, 37%) were purchased from VWR Chemicals. Dimethyl sulfoxide (DMSO ≥ 99.5%) and hydrogen peroxide (H_2_O_2_, 30% in water) were purchased from Carl Roth. Sulfuric acid (H_2_SO_4_, ≥95%) was purchased from Fisher Scientific (U.K.). Trimethylsilyl cellulose (TMSC, prepared by silylation of Avicel, DS_Si_ = 2.74, *M*_w_ = 181,000 g·mol^–1^, *M*_n_ = 30,400 g·mol^–1^, and PDI = 6.1, determined by size exclusion chromatography in chloroform) was purchased from TITK (Rudolstadt, Germany). Endo-1,4-β-d-xylanase from Neocallimastix patriciarum (cat. no. E-XYLNP; UNIPROT accession no. P29127; GH11) was purchased from Megazyme International (Bray, Ireland). All of the abovementioned chemicals were used as received. SPR metal sensor slides (CEN102Au) with a chromium adhesion layer of approximately 5 nm on glass and a gold coating of approximately 50 nm were obtained from Cenibra (Bramsche, Germany). Silicon wafers with a thickness of 675 ± 25 μm were purchased from Siegert Wafers (Aachen, Germany). Milli-Q water (resistivity = 18.2 MΩ·cm) from a Millipore water purification system (Millipore) was used for all experiments.

### Thin Film Preparation

SPR sensor slides were cleaned for 2 min with a piranha solution containing H_2_O_2_ (30 wt %)/H_2_SO_4_ (1:3 v/v), followed by rinsing with Milli-Q water for several minutes and subsequently dried in a nitrogen stream. Silicon wafers were cut into 1 × 1 cm^2^ pieces and cleaned prior to use by immersion in the acid piranha solution containing H_2_O_2_ (30 wt %)/H_2_SO_4_ (1:2 v/v) for 16 min, followed by rinsing with distilled water for several minutes and subsequently dried in a nitrogen stream. Xylan was dissolved in DMSO at different concentrations of 10 and 20 mg·mL^–1^ aided by activation with deionized water according to Schaubeder et al.^19^ Lignin was dissolved directly in DMSO at a concentration of 20 mg·mL^–1^. For the production of the blend films, lignin powder was added in the corresponding amounts to the predissolved xylan solution. All solutions were directly used for spin coating. TMSC was dissolved in chloroform at a concentration of 0.75 wt % in an ultrasonic bath (45 W) accompanied by heating (60 °C) for approximately 6 h. The solution was then filtered with a 0.45 μm poly(tetrafluoroethylene) (PTFE) filter, and the resulting solution (80 μL·cm^–2^) was deposited on the SPR sensor slides and subjected to spin coating (*t* = 60 s with a spinning speed of 4000 rpm and an acceleration of 2500 rpm·s^–1^). The TMSC films were regenerated for 14 min by exposing them to HCl vapor generated from a diluted HCl solution of 10%.^20^ Without further treatment, 80 μL·cm^–2^ of the corresponding xylan/lignin blend solution was spin-coated on the cellulose film (*t* = 60 s, 4000 rpm, 2500 rpm·s^–1^) according to a modified literature procedure.^21^

### Moisture Uptake

To determine the moisture uptake of all films, free-standing films were cast from solutions of Xyl, Xyl/Lig 2:1, and Xyl/Lig 1:1. The 2:1 Xyl/Lig and Lig solutions did not give homogeneous films and were therefore not used for the moisture uptake determinations. The caster films were prepared by pipetting 400 μL of the respective solution into round molds with a diameter of 2.5 cm and then dried in a drying oven at 70 °C for 1 h. The resulting films and the corresponding mixtures of xylan and lignin powders were conditioned for 24 h in a climate room at 23 °C and 50% relative humidity to determine gravimetrically the moisture uptake (*m*_moist_). The vessels used for the determination were first stored in a drying oven at 105 °C for 24 h and then in a desiccator to create the same conditions for the empty weight. All films and powders were then dried in a drying oven at 105 °C and stored in a desiccator to cool down. The weights of the dry films and powders (m_dry_) were then determined using a Sartorius precision balance (Germany). The moisture uptake of the films and powders was then calculated according to eq 1 and is expressed as a percentage. A duplicate determination was performed.
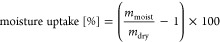
1

### Enzyme Solutions

Endo-1,4-β-xylanase (GH11, N. patriciarum) was diluted 1000-fold and 2000-fold (to 10 and 5 U·mL^–1^) in 100 mM sodium phosphate buffer (SPB), pH 6. One unit of xylanase activity is defined as the amount of enzyme required to release one μmol of xylose-reducing sugar equivalents per minute from wheat arabinoxylan (Megazyme, Bray, Ireland). The 10 U·mL^–1^ solution was used for the HS-AFM experiments and had a xylanase concentration of 12.5 μg·mL^–1^, where 100 μL of xylanase solution were added to 300 μL of sodium phosphate buffer directly during the measurement. The 5 U·mL^–1^ solution was used for the SPRS experiments and had a xylanase concentration of 6.25 μg·mL^–1^, where a total of 200 μL of xylanase solution was applied in the SPRS chamber with a flow rate of 25 μL·min^–1^. Two different batches of enzyme stock solutions were used for the HS-AFM and SPRS experiments, respectively.

### Multiparameter Surface Plasmon Resonance Spectroscopy

Real-time enzymatic degradation experiments were performed using an MP-SPR Navi 210 VASA from BioNavis Ltd. (Tampere, Finland) containing four lasers (λ = 670, 785, 850, and 980 nm) in measurement chamber A and two lasers (λ = 670 and 785 nm) in measurement chamber B. All measurements were performed at 25 °C using an angular scan range of 50–78° and a scan speed of 8°·s^–1^. The coated SPR sensor slides (glass substrate with an ∼5 nm thick chromium adhesion layer and an ∼50 nm thick gold layer) were first equilibrated by rinsing with sodium phosphate buffer (SPB) at a flow rate of 25 μL·min^–1^ for about 30 min. After equilibration, the abovementioned enzyme solution (xylanase at 5 U·mL^–1^) was injected into the system and allowed to adsorb/degrade for 8 min at a flow rate of 25 μL·min^–1^, followed by a washing step with SPB to rinse off the loosely bound material. For the experiments including BSA, 0.5 mg·mL^–1^ BSA was added to the buffer solution for equilibration and to the enzyme solution. Triplicates were performed for each film, resulting in a total of six measurements per experiment. BioNavis Dataviewer software was used for data processing. De Feijter equation (eq 2) was used to calculate the amount of digested xylan Γ (mg·m^–2^).^22^ The change in SPR angle ΔΘ (deg) is calculated by taking the average values from 10 min of the stabilized signal before and after the experiment was performed. The term *k*·d*p* (cm·°^–1^) can be considered constant for thin films (<100 nm) and can be calculated by calibrating the instrument by determining the decay wavelength ld. For the MP-SPR Navi 210A VASA used in this study, the k·dp value is about 1.90 × 10^–7^ cm·°^–1^ for the 785 nm laser in aqueous systems. The refractive index increment (d*n*/d*c*) of 0.158 cm^3^·g^–1^ was used as reported for xylan.^19^
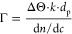
2

### High-Speed Atomic Force Microscopy Measurements

HS-AFM^23^ measurements were conducted using a tip-scan HS-AFM (PS-NEX, Research Institute of Biomolecular Metrology, Co., Ltd., Tsukuba, Japan).^24^ The system was operated in tapping mode for all measurements. The cantilevers were AC10 (Olympus, Tokyo, Japan) cantilevers with a carbon tip grown by electron deposition at the free end of the beam. After plasma etching, the tip radius is sharpened to typically 1 nm. The dimensions of the cantilevers are specified as follows: 9 μm long, 2 μm wide, and 130 nm thick, giving a nominal spring constant of 0.1 N m^–1^ and a resonance frequency of around 500 kHz. Images were typically recorded with a frame time of 12 s and a resolution of 200 × 200 pixels. All samples were prepared on Si wavers as described above and were glued to a microscopy glass slide (76 × 24 × 1 mm) by using nail polish directly prior to HS-AFM measurements. A 200 μL droplet of the SPB buffer solution was applied onto the glass slides to form a stable meniscus between the AFM scanner head and the sample, ensuring that the samples could be imaged stably for at least 2 h before the evaporated volume needed to be replenished. Before the addition of xylanase, the surface of the sample was characterized in SPB thoroughly at various scan sizes (1 × 1 μm^2^, 500 × 500 nm^2^, and 200 × 200 nm^2^). During the scanning, xylanase was added to the observation buffer, and the enzymatic degradation was monitored. After xylan was completely removed, the resulting surface was characterized again by using the aforementioned three scan sizes.

## Results and Discussion

### Preparation and Morphology of the Xylan/Lignin Composite Thin Films

**Figure 1:**
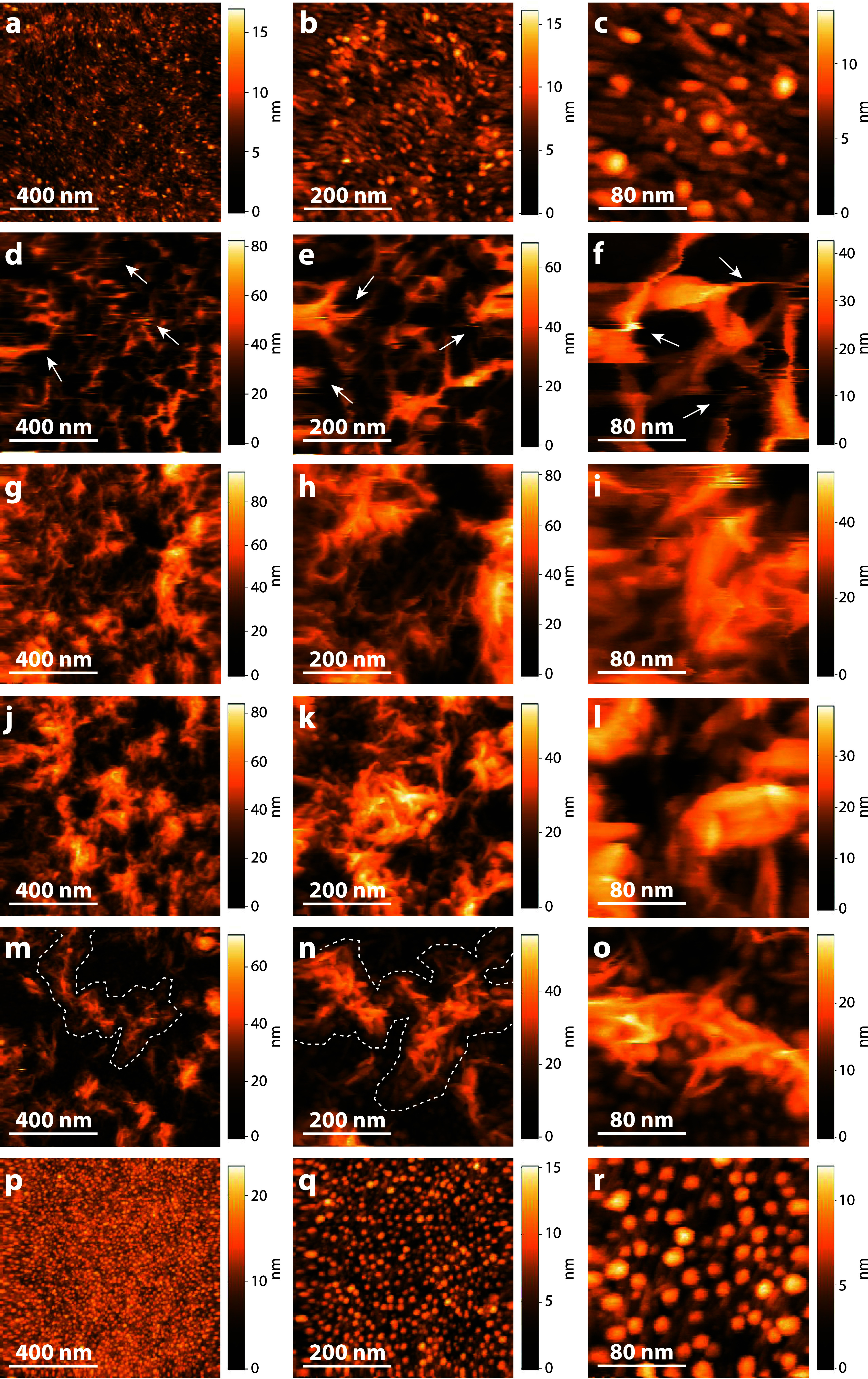
Height images of all films in sodium phosphate buffer recorded by HS-AFM measurements with a scan size of 1 × 1 μm^2^ (left column), 500 × 500 nm^2^ (middle column), and 200 × 200 nm^2^ (right column). (a–c) Cell, (d–f) Xyl (white arrows mark disturbances of the soft xylan by the oscillating tip); (g–i) Xyl/Lig 2:1, (j–l) Xyl/Lig 1:1, (m–o) Xyl/Lig 1:2 (xylan domains are marked by the dashed lines); and (p–r) Lig.

The first step was to create a simplified cell wall matrix in the form of thin films on different types of surfaces (Si and Au). Therefore, a base layer of cellulose (Cell) was deposited using the well-established trimethylsilyl cellulose route.^25^ On this base layer, mixtures of xylan and lignin were deposited in different ratios by spin coating (Xyl/Lig 2:1, 1:1, and 1:2) and compared to the pure components (Xyl, Lig). The morphology of the resulting films in sodium phosphate buffer (SPB) is shown in Figure 1. The cellulose films (Figure 1a–c) show a fibrillar surface structure with a preferred orientation. At lower resolutions and when measured in a dry state, these thin films appear to have spherical features,^19,25−27^ but at a higher resolution, the fibrillar structures of the cellulose thin films become apparent,^28^ which are more pronounced in liquid-state measurements. The diameter of these fibrils can be determined by measuring the height profile of at least three tightly packed, parallel fibrils using the width of the central fibril according to Figure S1. An average of 9.3 ± 0.9 nm fibril diameter (average of seven line profile measurements) was determined, which corresponds well with the smallest cellulose nanofibril subunit determined for cellulose thin films of 11 ± 2 nm.^29,30^ In addition, the HS-AFM images of Cell (Figure 1a–c) show regularly spaced higher spots with an average height of 8.4 ± 1.2 nm (average of 30 line profile measurements). These higher spots might be related to larger structures forming from the subunit nanofibrils. The Cell films are stable in the SPB and are not significantly affected by the force of the tip. In contrast, xylan swells strongly in solution and softens considerably compared to cellulose.^31^ Such AFM measurements are challenging^32^ as xylan can be easily distorted by the oscillating tip of the HS-AFM (see the horizontal streaks marked with white arrows in Figure 1d–f). To ensure that the tip does not displace xylan, the set point amplitude can be increased. Increasing the set point, while decreasing the scanning forces, will, however, negatively impact the resolution. This means that especially for soft samples, such as xylan, some disturbance of the surface is unavoidable. As the lignin content increases, the xylan appears to form a more stable, less soft structure, resulting in sharper AFM images with less distortions (Figure 1g–o; a video of the scanning is given in the Supporting Information, Video S1, showing a comparison of the mobility of the xylan without lignin and a stable imaging of the xylan in the presence of lignin). Moreover, xylan and lignin are not homogeneously distributed over the cellulose surface in the blend films. Rather, they form domains of lignin and xylan, the size of which depends on the ratio of the biopolymers. However, the boundaries of these domains are not exactly defined in the HS-AFM images. It appears that xylan interacts with lignin by forming domains on the lignin surface, which are best visible in the Xyl/Lig 1:2 blend films (marked by the dashed white lines in Figure 1m–o).

In general, the structure of a thin film consisting of two microscopically immiscible polymers, the standard case, in a common solvent is governed by wetting and solubility, leading to microphase separation. According to the literature, microphase separation during spin coating proceeds via the formation of two wetting layers and the collapse of their interface, causing the formation of lateral phase separation.^33^ Furthermore, wetting inversion may come into play, leading to monolayer thick coatings on the surface of the domains.^34,35^ This concept has also been observed for films consisting of lignocellulose polymers.^35^ In our system, this would result in a xylan–lignin matrix that is laterally phase separated and where potentially some sort of phase inversion might occur. Therefore, the xylan in the blend films might be less mobile, which could explain why the xylan in the lignin-containing films (Figure 1g–o) deforms less under the scanning forces than the pure Xyl films (Figure 1d–f). In addition, the xylan–lignin blends take up less water with increasing lignin content owing to the hydrophobic nature of lignin; hence, the xylan in the blends is less soft. For the pure xylan and lignin powders and the powder blends, a gravimetric decrease in moisture uptake was observed from +13.0% (Xyl) to +9.7% (Xyl/Lig 2:1), 8.4% (Xyl/Lig 1:1), 6.4% (Xyl/Lig 1:2), and 2.7% (Lig) (Table S1), showing that the xylan powder takes up almost three times more water than the used lignin. The same decreasing trend was observed for the moisture uptake of the free-standing films, from +11.2% (Xyl) to +9.0% (Xyl/Lig 2:1) and +7.1% (Xyl/Lig 1:1) (Table S1). The free-standing films adsorbed slightly less moisture than the powders due to their smaller surface area. A decreased swelling was also observed for regenerated fibers composed of cellulose, lignin, and xylan when exposed to water.^36^ The pure Lig films on cellulose show a homogeneous distribution of stable, spherical lignin clusters over the entire cellulose surface (Figure 1p–r). However, closer inspection reveals that the lignin particles are embedded in the amorphous fibrillar cellulose matrix. Rojo et al.^37^ proposed a model that describes lignin nanoparticles adhering to the cellulose nanofibrils and filling the voids between fibers, resulting in a similar pattern as to the thin films prepared.

When comparing the pure Lig films (Figure 1p–r) with the pure Xyl films (Figure 1d–f), it seems that the amount of xylan is higher than the amount of lignin, even though the applied amount and concentrations are the same. This is attributable to the better wettability of xylan on cellulose compared to lignin. A decrease in wettability with increasing lignin content was also observed for regenerated fibers composed of cellulose and lignin.^36^

### Enzymatic Degradation

**Figure 2:**
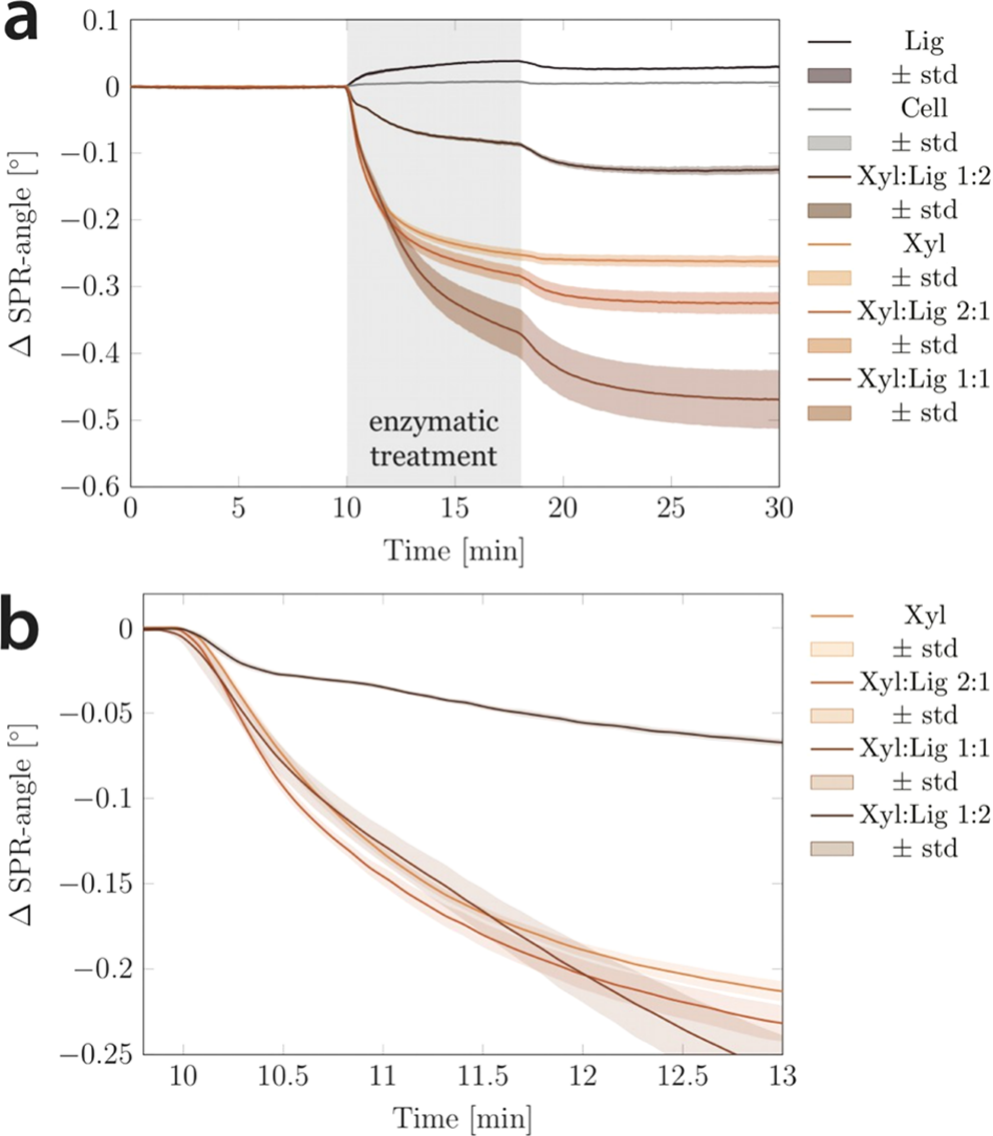
(a) SPRS curves of the treatment of all films (Cell, Xyl, Xyl/Lig 2:1, Xyl/Lig 1:1, Xyl/Lig 1:2, and Lig) with an endoxylanase. (b) Zoom into the SPRS curves of the beginning of the enzymatic degradation of the Xyl and all blend films (Xyl/Lig 2:1, Xyl/Lig 1:1, and Xyl/Lig 1:2).

SPRS is capable of monitoring in real-time *in situ* enzymatic degradation on surfaces. The SPR curves (Figure 2a) reveal that the xylanase adsorbs to the Cell film but only to a minor extent and desorbs again upon washing. However, a clear adsorption of the xylanase to the Lig film is evident, where the xylanase does not fully desorb during washing. This indicates weak binding of xylanase to the lignin surface (0.31 ± 0.02 mg·m^–2^). However, the baseline of Lig is not stable during washing after the experiment, indicating that the system is not in equilibrium. Hence, desorption of the xylanase from the lignin surface might still take place. Weak and nonspecific interactions between a different GH11 xylanase and an oligomeric lignin analogue have also been described in the literature.^34^

Interestingly, the enzymatic treatment of the Xyl/Lig 2:1 (−3.89 ± 0.20 mg·m^–2^) and Xyl/Lig 1:1 (−5.61 ± 0.55 mg·m^–2^) blend films resulted in a higher degradation compared to the pure Xyl (−3.16 ± 0.11 mg·m^–2^) films, and it is not immediately evident why. Even though the pure Xyl films and the blends were coated from solutions containing the same amount of xylan, more xylan may attach to the cellulose surface in the presence of lignin. The amounts of xylan deposited on each film were analyzed by the height distributions of the HS-AFM images, which can be fitted with a simple, bimodal, or trimodal Gaussian function. A detailed description of the fitting procedure is provided in the Supporting Information, and the fitted histograms of all films are shown in Figure S3. In short, one Gaussian height distribution is expected per component on the films, such as cellulose, xylan, and lignin. Hence, for evaluating the cellulose average height distribution, a simple Gaussian function is used, while for lignin on cellulose, a bimodal Gaussian function is used for fitting the height profile. Because of the strong swelling capacity of xylan, the fitting with the bimodal Gaussian function did not provide satisfactory results (Figure S2); hence, the xylan on the cellulose film is fitted with a trimodal Gaussian function where we can divide the xylan film into two regions: the region that is directly in contact with the cellulose (bottom) and the strongly swollen, soft xylan that can be easily moved with the AFM tip in the liquid (top). Layer heights can be assigned to the individual components of the blend thin films, such as cellulose, lignin, and xylan, by subtracting the average height of the height profile of the cellulose layer from the height of the corresponding xylan or lignin layer (Table 1). Starting from the simplest films, the average height for the cellulose layer is around 8 nm, which was determined for all films with slight deviations for the Xyl films. Moreover, the average cellulose height could not be detected for the Xyl/Lig 1:1 films, probably because these films contain more xylan and lignin covering the cellulose layer completely. Two height distributions were determined for the Lig films, resulting in an estimated lignin layer thickness of only about 2 nm. As shown, the lignin clusters are embedded in the cellulose fibrils, so the overall average layer height is low. However, when xylan is present, the average height of the lignin layer increases to about 14.3 ± 1.4 nm for the Xyl/Lig 1:2 films and to about 24.9 ± 4.9 nm for the Xyl/Lig 1:1 films. For the Xyl/Lig 2:1 films, it is assumed that the determined average height of 33.2 ± 5.6 nm corresponds to a mixture of xylan and lignin that is in touch with the cellulose surface, with a layer of softer xylan on top. To obtain an estimate of the xylan layer thickness of all films, the average height of the lignin or cellulose layer was subtracted from the average xylan height (Table 2; layer thickness xylan_HS-AFM_). However, as can be seen from the standard deviations, these layer thicknesses are not exact, and the values should be regarded as estimates. Nevertheless, these values agree well with the degradation extent quantified using SPRS and confirm the higher degradation of the Xyl/Lig 1:1 film compared to the pure Xyl film (Table 2).

**Table 1:** Estimated Average Layer Heights of the Main Three Components, Namely Cellulose, Lignin, and Xylan on All Films Determined by HS-AFM Height Distribution Analysis of 1 × 1 μm^2^ Images by Gaussian Fitting^a^

	cell	Lig	Xyl	Xyl/Lig 2:1	Xyl/Lig 1:1	Xyl/Lig 1:2
cell	8.4 ± 0.1	7.9 ± 1.0	13.7 ± 0.8	7.8 ± 2.7		8.7 ± 0.4
Lig		9.7 ± 1.2		33.2 ± 5.6	24.9 ± 4.9	14.3 ± 1.4
Xyl_bottom_			23.1 ± 1.2		36.4 ± 4.8	22.9 ± 2.7
Xyl_top_			31.4 ± 6.0	48.8 ± 5.6	44.9 ± 6.4	

a All values are given in nm.

**Table 2:** Degraded Xylan Amounts from the Different Films Using Xylanases Determined by SPRS Experiments Given in Surface Concentration Γ and Corresponding Layer Thickness Reduction Calculated with a Xylan Layer Density of 1.2 g·cm^–3^, Estimated Xylan Layer Thicknesses Determined by Height Distribution Analysis of the HS-AFM Measurements, and Slopes of the Initial Linear Degradation (Marked by the Dashed Lines in Figure S4b)

film	Γ [mg·m^–2^]	layer thickness reduction_SPRS_ [nm]	layer thickness xylan_HS-AFM_ [nm]	slope of initial degradation [°·min^–1^]
Xyl	–3.16 ± 0.11	–2.6 ± 0.1	17.7 ± 6.8	–0.163
Xyl/Lig 2:1	–3.89 ± 0.20	–3.2 ± 0.2	15.6 ± 11.2	–0.203
Xyl/Lig 1:1	–5.61 ± 0.55	–4.7 ± 0.5	20.0 ± 11.3	–0.161
Xyl/Lig 1:2	–1.47 ± 0.08	–1.2 ± 0.1	8.6 ± 4.1	–0.078
Lig	+0.31 ± 0.02	+0.3 ± 0.02		

The first row shows the average height of the cellulose layer of all films. The second row shows the average height of the lignin layer; hence, for the lignin layer thickness determination, the average height of the cellulose layer needs to be subtracted from the average height of the lignin layer for each film. The same applies to the xylan average layer heights.

On the Xyl/Lig 1:2 (−1.47 ± 0.08 mg·m^–2^) films, less xylan was degraded compared to the pure Xyl films (Figure 2 and Table 1). This is a result of the lower xylan concentration used for spin coating and a thinner xylan layer of 8.6 ± 4.1 nm compared to the other samples, as shown by height distribution analysis. Hence, less xylan is available for degradation on this system. Moreover, the SPR curve also exhibits a slightly different behavior than for the other films. The degradation can be divided into two phases. First, the SPR curve begins to drop exponentially after the addition of xylanase but soon slows down after 30 s. At this stage, there is probably not much xylan left for degradation, so the adsorption of the xylanase to the lignin is more significant, as evidenced by an increase in the SPR angle, followed by slow degradation of the remaining xylan. After initiation of the washing, a stronger decrease in SPR angle is observed again compared to the Xyl/Lig 2:1 films, indicating that xylanases are still present on the lignin surface and are continuously detaching. The amounts of degraded xylan are expressed in mg·m^–2^ and in nm, and the estimated values for the xylan layer thickness determined by HS-AFM are shown in Table 2. To further investigate the contribution of lignin, the Xyl/Lig 2:1 films were also treated with xylanase in the presence of BSA, which was identified as a lignin-blocking additive.^38^ The comparison of the SPR curves of the films treated without and with BSA (Figure S4a) shows no sudden decrease in the SPR angle at the initiation of the washing step when BSA was present. Therefore, this decrease can indeed be correlated to the xylanase desorbing from the lignin, and the continuous desorption of xylanase from lignin is the reason for the baseline not stabilizing after the experiment during subsequent washing.

Conclusions about the degradation kinetics can be drawn from the initial decrease in the slope of the SPR angle. The higher the initial slope, the faster the degradation kinetics. The slope from the initial linear degradation (dashed region in Figure S4b) from 10.1 to 10.25 min was determined (Table 2). A zoomed-in view of the beginning of the degradation region is shown in Figure 2b, where it is evident that the degradation kinetics are fastest for the Xyl/Lig 2:1 films (−0.203°·min^–1^). This is surprising as less degradation was expected in the presence of lignin. The second fastest degradation kinetics are observed for the Xyl films (−0.163°·min^–1^), while slightly slower initial degradation kinetics are observed for the Xyl/Lig 1:1 films (−0.161°·min^–1^) and much slower kinetics are observed for the Xyl/Lig 1:2 films (−0.078°·min^–1^). Thus, there appears to be a threshold in lignin content for allowing the digestion of xylan, and although xylanase is active on lignin (evidenced by SPR angle changes), the action is significantly slower at higher lignin contents. One plausible explanation for the faster degradation of the Xyl/Lig 2:1 films is the change in the xylan film structure from the Xyl to the blend films. The xylan in the pure Xyl films exhibited less stability to the HS-AFM tip than the xylan in the blend films and appeared to be softer (Figure 1). As the xylan in the Xyl films takes up more water than the xylan in the blend films, this softer xylan could be less accessible to the xylanase. Water sorption studies on chitosan, cellulose, and alginate showed that water clusters formed around functional groups.^36^ Since the used xylanase requires three consecutive unsubstituted xylose units and cleaves the backbone one unit before a methylglucuronic acid side chain residue,^39^ it is speculated that higher adsorption of water molecules to the xylan could hinder the adsorption of the xylanase and hence the cleavage. This would mean that with less water adsorption, the xylan becomes more accessible to the xylanase and thus enhances the degradation even when the lignin content increases. At a certain point, however, the nonspecific binding of xylanase to lignin can exceed this effect and hinder degradation.

### Morphology of Blend Films after Enzymatic Degradation

**Figure 3:**
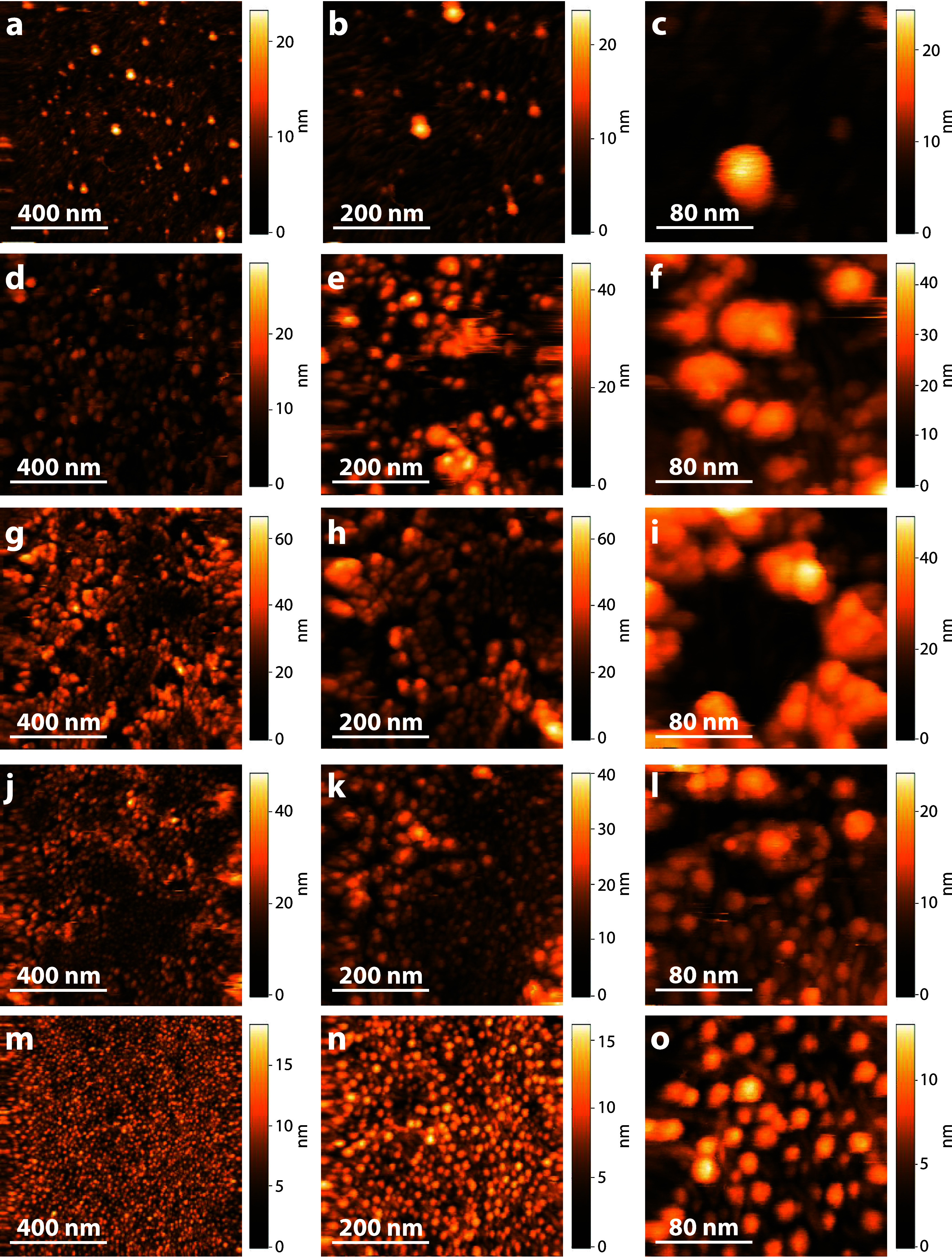
Height images of all films in sodium phosphate buffer determined by HS-AFM measurements with a scan size of 1 × 1 μm^2^ (left column), 500 × 500 nm^2^ (middle column), and 200 × 200 nm^2^ (right column) after enzymatic degradation with a xylanase. (a–c) Xyl, (d–f) Xyl/Lig 2:1, (g–i) Xyl/Lig 1:1, (j–l) Xyl/Lig 1:2, and (m–o) Lig.

**Figure 4:**
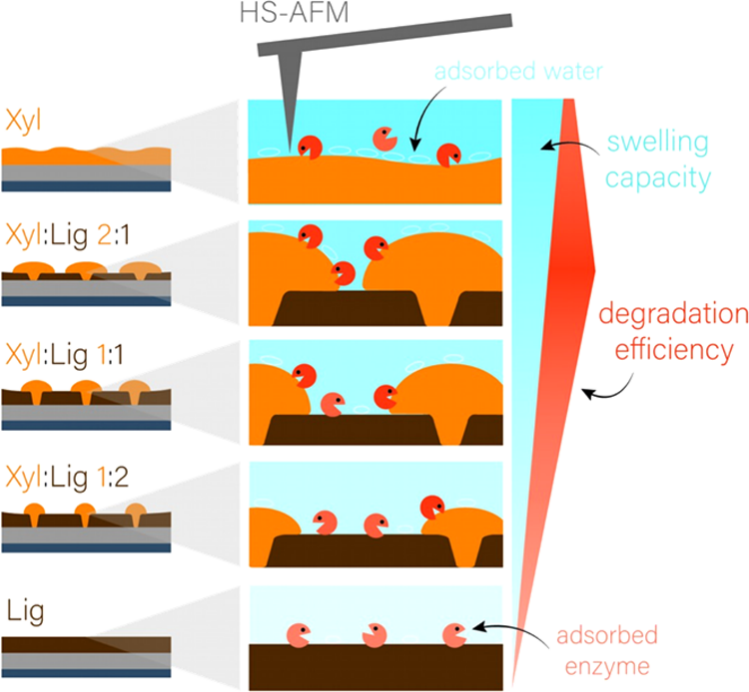
Mechanistic diagram showing the differences in film formation and corresponding differences in enzymatic degradation efficiency. Swelling capacity increases from Lig to Xyl (bottom to top), leading to adsorption of water on the xylan surface. With an increasing lignin content, more enzymes are adsorbed to the surface, decreasing the overall efficiency. Hence, the highest degradation efficiency was observed for the Xyl/Lig 2:1 films.

To investigate the changes in morphology after xylan breakdown, we also tracked the degradation by HS-AFM. As mentioned previously, scanning forces could distort the soft xylan layer, which could be confused with degradation even when no xylanase was present. When carefully adjusting the set point amplitude, stable imaging was possible before the addition of xylanase. However, after xylanase was added, even the highest stable amplitude set point could not prevent xylan from being removed from the surface. While such videos do not show undisturbed degradation and cannot be used to infer kinetics of the process, they show that there is an almost immediate effect of the enzymes on xylan (see Supporting Information, Video S2: Xyl, Video S3: Xyl/Lig 2:1, Video S4: Xyl/Lig 1:1, Video S5: Xyl/Lig 1:2). Nevertheless, the morphologies of the films after enzymatic degradation are clearly apparent. The height images (Figure 3) demonstrate that the xylan films’ morphologies disappeared completely after enzymatic degradation. Further, the cellulose surface was at least partially visible on all films. A reason for the discrepancy in the SPRS results could be the different experiment durations (shorter degradation time in SPRS experiments), the influence of the flow rate of the SPRS, and the influence of the tip of the HS-AFM. In the pure Xyl films (Figure 3a–c), aggregates of 11.5 ± 3.3 nm in height (average of 30 aggregates, measured by line profile analysis) were formed after degradation, which are probably shorter-chain, degraded xylan aggregates, since the used GH11 xylanase randomly cleaves the backbone and releases shorter xylan chains.^37^ Moreover, after enzymatic degradation, it was evident that xylan was not only located on the lignin, but that these xylan domains were also in contact with the cellulose, leaving cracks and deformations in the lignin layer (Figure 3d–l). In addition, in the presence of xylan, lignin appears to form larger aggregates near the xylan domains than the pure lignin on cellulose, indicating a stronger interplay between lignin and xylan than between lignin and cellulose. Treatment of the pure Lig films with xylanase (Figure 3m–o) did not result in any change in film morphology. However, during treatment, the xylanase is adsorbed and desorbed to and from the lignin surface (Supporting Information, Video S6, xylanases are indicated by blue arrows), but there is no clear evidence of nonreversible binding of xylanases. It is assumed that these so-called nonproductive interactions between enzymes and lignin are not conducive to degradation, which explains the slower degradation kinetics at higher lignin contents. As shown in Figure 1, the lignin clusters in the Lig films are homogeneously distributed over the cellulose surface and interact with the cellulose fibrils. This is also observed in the Xyl/Lig 1:2 blend films after degradation. However, in the Xyl/Lig 1:1 and Xyl/Lig 2:1 blend films, the lignin appears to preferentially interact with xylan rather than cellulose and forms a separate layer, which is also demonstrated by height distribution analysis (Figure S3 and Table 1). A mechanistic diagram of the film formation of the different films and the treatment with a xylanase is depicted in Figure 4. As the lignin content increases, the swelling capacity of the films decreases, but the adsorption of xylanases on the lignin surface also increases, resulting in a decrease in degradation efficiency, while higher swelling leads to adsorbed water, which partially hinders the accessibility of enzymes. The fastest degradation efficiency was observed for the Xyl/Lig 2:1 films, representing a trade-off of the described effects.

## Conclusions

The investigation of the resilience of xylan to enzymatic degradation when it is in a matrix with the selected lignin by HS-AFM revealed that xylan and lignin form distinct domains on cellulose with the amount of xylan present on the films depending on the concentration of lignin. In addition, the xylan structure changed depending on whether lignin was present and led to a softer xylan domain when no lignin was present. The presence of some lignin in the blend films (Xyl/Lig 2:1) resulted in faster kinetics of xylan degradation compared with degradation of the pure Xyl films. It is speculated that the higher water adsorption of the softer xylan domain makes it more difficult for the xylanase to access and, thus, reduces the degradation rate. In contrast, faster degradation would be expected with a decrease in water uptake and thus also an increase in lignin content. However, when the lignin content of the blend films increases, the kinetics slow considerably, which can be related to the weak interactions (adsorption/desorption) between the xylanase and the lignin, making it unavailable for successful degradation. So, to answer the question from the title: wood xylan is not resilient to enzymatic hydrolysis by an endoxylanase when it is in a matrix with the selected lignin in the chosen model system. Under these conditions, enzymatic degradation even benefits from small amounts of lignin in the films; however, for films containing larger amounts of lignin, degradation was slowed down considerably. While the findings of degradation that this model system demonstrates are consistent, making conclusions regarding the native cell wall remain veiled due to the use of isolated lignins and xylans. The lignin we have employed in this study is a technical lignin, which has undergone depolymerization and, to some extent, also chemical modification during sulfate cooking. The same applies to the xylan, which has been obtained from an industrial process and, hence, has a structure different from the native state. However, the combination of these materials made sense from two viewpoints: (i) biotechnological valorization of xylan in a biorefinery where often also technical lignins are present and (ii) good solubility of both components in a common solvent such as DMSO enabling high reproducibility in film preparation and subsequent enzymatic degradation using surface sensitive techniques. We foresee that using the methodology established here and extending the matrix component selection to various isolates, such as milled wood lignin and lignin-carbohydrate complexes, will be important for progressing the knowledge of wood component matrix recalcitrance toward enzymatic activity.

## Supporting Information Available

Detailed description of the Gaussian fitting, further details about the SPRS measurements, and supporting HS-AFM images of all untreated films in SPB with a scan size of 500 × 500 nm^2^ (Table S1 and Figures S1–S5) (PDF)
Comparison of scanning of xylan in the absence and in the presence of lignin (Video S1) (MP4)
Enzymatic degradation of xylan from Xyl film (Video S2) (MP4)
Enzymatic degradation of xylan from Xyl/Lig 2:1 film (Video S3) (MP4)
Enzymatic degradation of xylan from Xyl/Lig 1:1 film (Video S4) (MP4)
Enzymatic degradation of xylan from Xyl/Lig 1:2 film (Video S5) (MP4)
Xylanase adsorbing on and desorbing from the Lig film (Video S6) (MP4)

## Notes

The author(s) used DeepL Translator and GPT-3.5 during the preparation of this work to find appropriate synonyms, phrases, and linking text blocks to better explain the subject and make the text more coherent. After using this tool/service, the author(s) reviewed and edited the content as needed, and they take full responsibility for the content of the publication.

## Abbreviations

BSA: bovine serum albumin
HS-AFM: high-speed atomic force microscopy
SPB: sodium phosphate buffer
SPRS: surface plasmon resonance spectroscopy
