# Supplementary material for: How Resilient is Wood Xylan to Enzymatic Degradation in a Matrix with Kraft Lignin?
Source: Biomacromolecules. 2024 May 15;25(6):3532–41. doi: 10.1021/acs.biomac.4c00185 (PMC11170953; doi:10.1021/acs.biomac.4c00185)
Supplement: Supplementary file 1 — bm4c00185_si_001.pdf [file bm4c00185_si_001.pdf]

Supporting Information

How resilient is wood xylan to enzymatic degradation in a matrix with kraft lignin?

Jana B. Schaubeder ◊, Christian Ganser ‡, Tiina Nypelö ∅*, Takayuki Uchihashi ‡°, Stefan Spirk ◊**

◊ Graz University of Technology, Institute of Bioproducts and Paper Technology (BPTI), Inffeldgasse 23, 8010 Graz, Austria

‡ National Institutes of Natural Sciences, Exploratory Research Center on Life and Living Systems, 5-1 Higashiyama, Myodaiji, 444-8787 Okazaki, Japan

∅ Aalto University, Department of Bioproducts and Biosystems, Vuorimiehentie 1, 02150 Espoo, Finland and Chalmers University of Technology, Kemivägen 10, 41296 Gothenburg, Sweden

° Nagoya University, Department of Physics, Chikusa-ku, Furo-cho, 464-8602 Nagoya, Japan

* To whom correspondence should be addressed. E-Mail address: cganser@ims.ac.jp (C. Ganser), tiina.nypelo@aalto.fi (T. Nypelö), and stefan.spirk@tugraz.at (S. Spirk)

1. Cellulose fibril size, moisture uptake, and distribution in height profiles

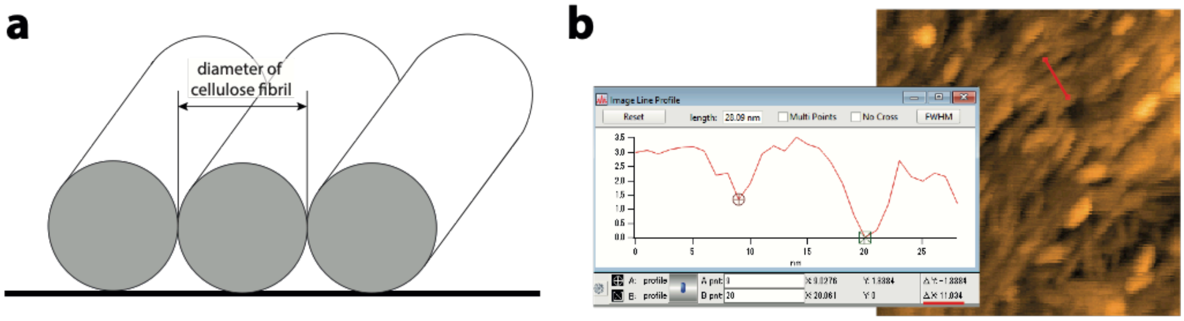

Figure S1: a) Scheme for determining the diameter of cellulose fibrils. b) Exemplary height profile analysis of an HS-AFM image in sodium phosphate buffer with a scan size of 200 × 200 nm^2 to determine the diameter of the cellulose fibrils.

Moisture uptake

Table S1. Determined moist and dry weights of xylan and lignin powders and powder mixtures and calculated moisture uptake in %. A duplicate determination was performed. Corresponding weights of free-standing films are given in brackets.

Powder (film)	m_{moist} [g]	m_{dry} [g]	Moisture uptake [%]	Average moisture uptake [%]
Xyl powder (Xyl film)	0.01940 (0.00829)	0.01718 (0.00756)	+ 12.9 (9.7)	13.0 (11.2)
	0.01807 (0.00815)	0.01597 (0.00723)	+ 13.1 (12.7)	
Xyl:Lig 2:1 powder (Xyl:Lig 2:1 film)	0.02949 (0.01291)	0.02670 (0.01190)	+ 10.4 (8.5)	9.7 (9.0)
	0.02763 (0.01264)	0.02535 (0.01155)	+ 9.0 (9.4)	
Xyl:Lig 1:1 powder (Xyl:Lig 1:1 film)	0.03962 (0.01713)	0.03621 (0.01592)	+ 9.4 (7.6)	8.4 (7.1)
	0.03765 (0.01505)	0.03508 (0.01411)	+ 7.3 (6.7)	
Xyl:Lig 1:2 powder	0.02854	0.02656	+ 7.5	6.4
	0.02681	0.02546	+ 5.3	
Lig powder	0.01586	0.01516	+ 4.6	2.7
	0.01382	0.01370	+ 0.9	

Fitting of height distributions

To evaluate layer thicknesses of the individual films and the corresponding amounts of xylan and lignin, the height distributions obtained from HS-AFM measurements were fitted with a simple, a bimodal, or a trimodal Gauss function, depending on the expected number of layers, *e.g.*, the pure cellulose film is fitted with a simple Gaussian function, as only one layer is expected. The **Lig** film (lignin on cellulose) is fitted with a bimodal Gaussian function, as two height distributions are expected for this film, one for the cellulose and one for the lignin. For the xylan film, a trimodal Gaussian function was used for fitting, as fitting with the bimodal Gaussian function did not provide satisfactory results (Fig. S2). This can be explained by the strong swelling of xylan, and we can divide the xylan films into two regions: the region that is directly in contact with the cellulose (bottom), and the strongly swollen xylan that can be easily moved with the AFM tip in the liquid (top). All blend films were fitted with the trimodal Gauss function, as three height distributions (for xylan, lignin, and cellulose) are expected. The histograms were exported using Falcon Viewer (Igor Pro 9 software) and the fitting was performed with Python v3.8.8. For each film, three different 1x1 μm^2 images were fitted and the average of the identified peaks was calculated. The fitted histograms of all films are shown in Fig. S3.

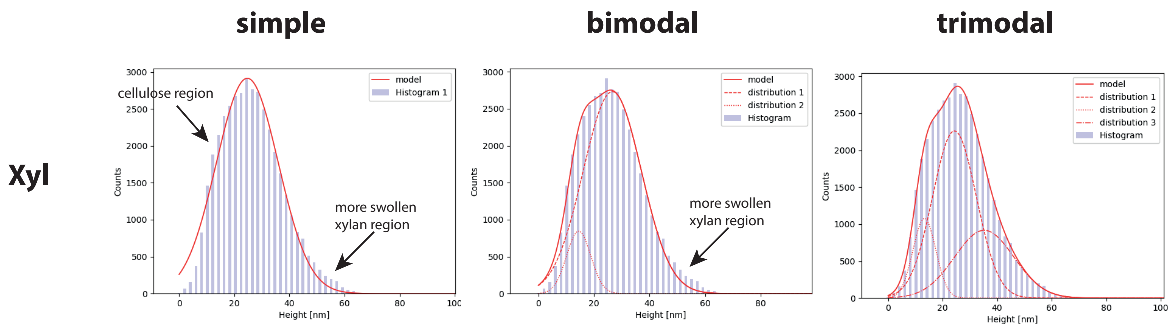

Figure S2: Height distribution of the Xyl film determined by HS-AFM in sodium phosphate buffer with a scan size of 1 × 1 μm^2. The height distribution was fitted by a simple (left), bimodal (middle), or trimodal Gaussian (right) function. Distribution characteristics that are not adequately represented by the corresponding fitting function are marked with a black arrow.

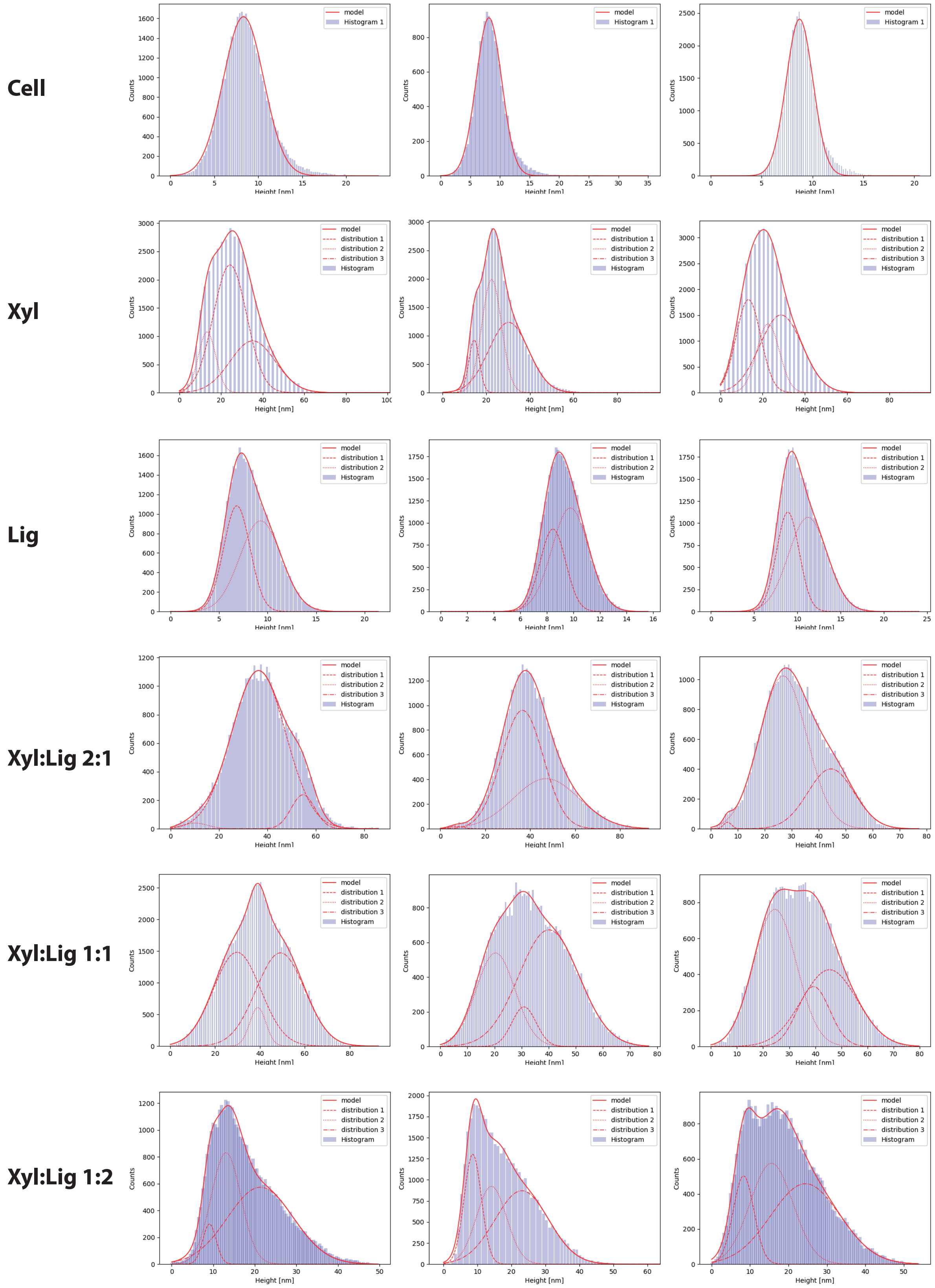

Figure S3: Height distributions of all films determined by HS-AFM in sodium phosphate buffer with a scan size of 1 × 1 µm^2. The distributions were obtained by a simple (Cell), bimodal (Lig), or trimodal (Xyl, Xyl:Lig 2:1, Xyl:Lig 1:1, Xyl:Lig 1:2) Gaussian fit of the histrograms. The average of the three

fitted peak means per film was calculated and related to the cellulose, xylan, or lignin layer. The film thickness for xylan and lignin were then determined by subtracting the cellulose average height from the calculated average height of the corresponding layer.

2. Further details of SPRS measurements

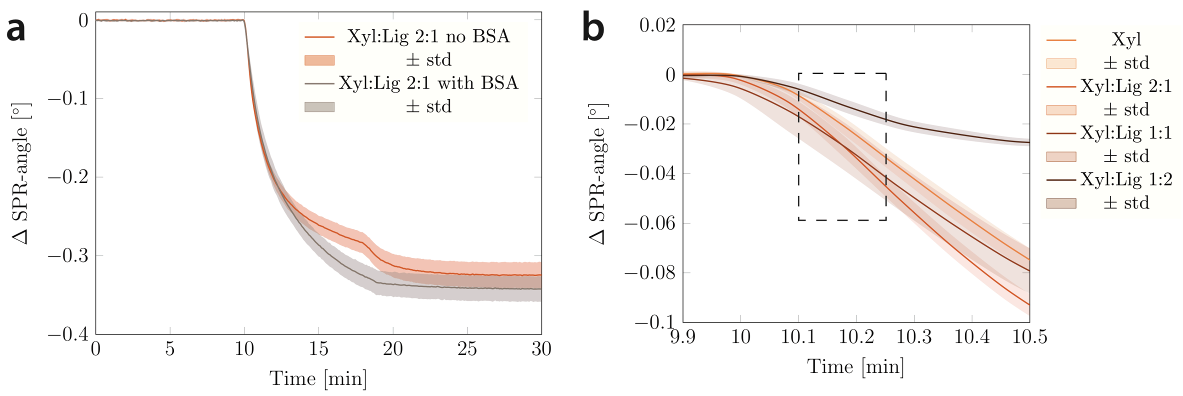

Figure S4: a) Comparison the Xyl:Lig 2:1 film treated with a xylanase without and with the presence of 0.5 mg·ml^{-1} *BSA and b) zoom into the initial decrease in SPR-angle (dashed line shows the region used to determine the slope of the initial degradation).*

3. HS-AFM images of all films

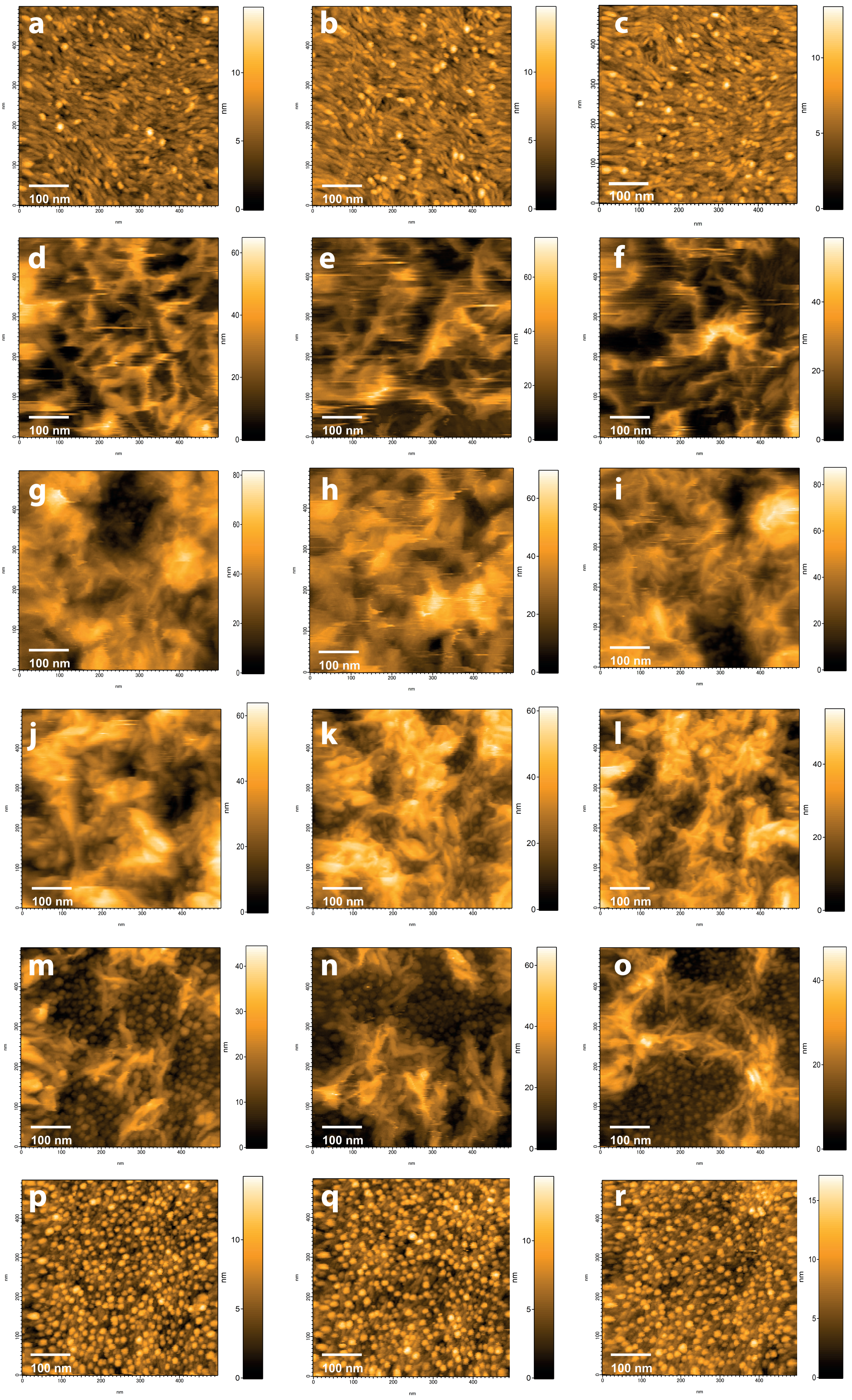
a
b
c
d
e
f
g
h
i
j
k
l
m
n
o
p
q
r
100 nm
nm

Figure S5: Supporting HS-AFM images of different films with a scan size of 500 × 500 nm^2 of a-c) cellulose, d-f) Xyl, g-i) Xyl:Lig 2:1, j-l) Xyl:Lig 1:1, m-o) Xyl:Lig 1:2, and p-r) Lig. Representative images were selected for the main manuscript.
